# Harnessing agricultural microbiomes for human pathogen control

**DOI:** 10.1038/s43705-022-00127-2

**Published:** 2022-05-19

**Authors:** Fiona P. Brennan, Beatrix W. Alsanius, Ana Allende, Catherine M. Burgess, Helena Moreira, Gro S. Johannessen, Paula M. L. Castro, Mieke Uyttendaele, Pilar Truchado, Nicola J. Holden

**Affiliations:** 1 Teagasc, Department of Environment, Soils and Landuse, Johnstown Castle, Wexford, Ireland; 2 Department of Biosystems and Technology; Microbial Horticulture Unit, Swedish University of Agricultural Sciences, Alnarp, Sweden; 3 Food Safety and Quality Group, CEBAS-CSIC, Campus Universitario de Espinardo, Murcia, Spain; 4 Food Safety Department, Teagasc Food Research Centre, Ashtown, Dublin, Ireland; 5 Universidade Católica Portuguesa, CBQF—Centro de Biotecnologia e Química Fina—Laboratório Associado, Escola Superior de Biotecnologia, Porto, Portugal; 6 Section for Food Safety and Animal Health Research, Norwegian Veterinary Institute, Oslo, Norway; 7 Department of Food Technology, Food Safety and Health, Faculty of Bioscience Engineering, Ghent University, Coupure links 653, Ghent, Belgium; 8 James Hutton Institute, Dundee, UK & SRUC, Aberdeen, UK

**Keywords:** Environmental microbiology, Microbial ecology

## Introduction

Plant microbial communities comprise a complex network of microorganisms present on surface and internal tissues, which interact with the host plant and the environment. Each plant compartment represents a microhabitat characterised by specific conditions that support distinct microbial communities. Whilst the plant microbiota is predominately beneficial or benign in nature, plant pathogens and, to a lesser extent, human pathogens, can be naturally present in the microbiome or become incorporated into the community. For edible crops, human pathogens represent a health risk and are increasingly responsible for foodborne disease, with fresh produce accounting for over 1/3 of all foodborne outbreaks in some countries. Growing evidence that certain human pathogens are well adapted to occupying plant niches necessitates a shift in thinking regarding their ecological range and should be incorporated within control strategies. In this article we argue that it is possible, through agricultural management practices, to enhance suppression of human pathogens by microbiota associated with horticultural crops. We examine the environmental fitness of these pathogens and the ecology of suppressive interactions, explore the knowledge gaps and approaches needed to fill these, and provide our perspectives on the potential to harness agricultural microbiomes to reduce the risk of disease transmission into the food-chain.

## Reassessing ecological ranges of pathogens

Shigatoxigenic *Escherichia coli* (STEC), *Salmonella enterica* and *Listeria monocytogenes* are the predominant cause of foodborne outbreaks in edible crops, attributed for >80% of cases [1]. While the latter is native to soil and considered a saprophyte, the former two pathogens are zoonotic. The primary reservoir of zoonotic pathogens are animals, including livestock, so they have traditionally been associated with meat and poultry food items. However, a notable outbreak in the USA & Canada of STEC associated with bagged baby spinach occurred in 2006, changing our perception of what was known as ‘the burger bug’ [2]. The outbreak resulted from contamination of spinach fields by wildlife [3] and the ability of the pathogen to spread over long-distance in watercourses [4], leading to 206 reported cases of disease with three fatalities. This led to step-changes in our understanding of the transmission of zoonotic pathogens via secondary hosts and habitats, from molecular mechanisms to demonstrated persistence and alternative metabolic pathways [5–7], to assessment of risk factors impacting spinach colonisation [8]. It also led to major changes in agricultural management including around irrigation water quality, organic amendments and avoidance of livestock contamination. Yet, the problems remain. For example, outbreaks of STEC associated with romaine lettuce have occurred repeatedly from the same genetic isolate in the same area over multiple years [9]. Further, there are indications that some agricultural measures implemented to protect consumers, such as removal of natural habitats or reduction in the use of organic amendments may be exacerbating the issue through a reduction in pathogen suppression [10, 11].

Although zoonoses like STEC and *S. enterica* are normally considered as adapted to warmed-blooded animals, they are physiologically adept and evolved to cope with a range of physio-chemico environments. Their mosaic genomes are littered with genetic elements orthologous to plant-adapted microbes, from inevitable horizontal gene transfer and recombination [12]. They are metabolically diverse, able to grow on a vast array of substrates including plant-derived, whether as partially digested material in animal guts or directly on plant hosts [13]. As mesophiles, their growth range spans conditions found in a variety of non-animal environments. They have competitive armouries from Type VI secretion systems [14] to antibiotic efflux pumps [15] and they form protective biofilms [16]. Only a handful of studies have examined growth rates in plants for any microbe, but zoonotic pathogens appear to be in line with native plant colonising bacteria [17]. They enter quiescent states, like viable-but-non-culturable, enabling long-term persistence before resuscitation under more favourable conditions. Although growth rates apparently reduce from inoculated plant experiments, this is often due to a high starting number that reduces to a more stable steady-state, and/or a consequence of culturability [18]. Environmental sampling has detected *E. coli* and *S. enterica* from a range of non-animal sources, and plant inoculation experiments reveal uncanny similarities to their plant-adapted taxonomic cousins in the Enterobacteriaceae [12, 19]. As such, it should come as no surprise that they can colonise plants, even if only transiently, and are transmitted by crop plants into the food chain. Generally, human pathogen cultural viability within soil, water or crops decreases over time, within a scale of weeks-months. This is within the growing cycle of crops associated with outbreaks; thus these can still become sufficiently established to cause food safety risks [20, 21].

The broad environmental fitness of human pathogens means that their capacity to be incorporated into plant microbiomes is unlikely to be restricted to horticultural crops and may occur wherever the opportunity arises. However, horticultural systems, some of which adopt management approaches that reduce system complexity (e.g. through the use of artificial growing substrates and extensive use of inorganic fertilisers or chemical inputs), and thus reduce microbial diversity, may be more vulnerable to successful pathogen establishment. There are also indications of plant specific differences, with the plant tissue type, the plant species and even plant genotype, strongly influencing the capacity to persist [22, 23]. Whatever variations in colonisation potential and plant defence exist across the plant kingdom, edible crops represent the greatest risk of transmission to human hosts due to their direct consumption. Intensive growing operations, complex distribution networks, and wide scale irrigation requirements within crops, which characterise much of modern production, can result in larger scale outbreaks, where contamination occurs. The majority of produce outbreaks are associated with ready-to-eat crops, which are inherently higher risk, but large outbreaks have also been associated with contamination of vegetables typically cooked [24]. Thus, human pathogens may be viewed as opportunists constituting a generic risk, associated with plant food production systems that requires consideration of pathogen growth in plants as a control target. With human pathogen contamination of produce now representing a serious and growing threat we need to completely rethink our preconceptions about zoonotic pathogens and the implications of their ability to persist out-with animal hosts. A key question is how we use existing knowledge, including adoption of concepts previously restricted to plant pathology, to mitigate the problem. We propose that knowledge of the ecological interactions of the plant holobiont (the plant and its associated microbiota), and manipulation through agricultural management practices present a very real solution for control. As such, we need to consider how the components of the plant holobiont impact on pathogen behaviour, and what can be feasibly manipulated.

## Shaping agricultural microbial communities

**Fig. 1:**
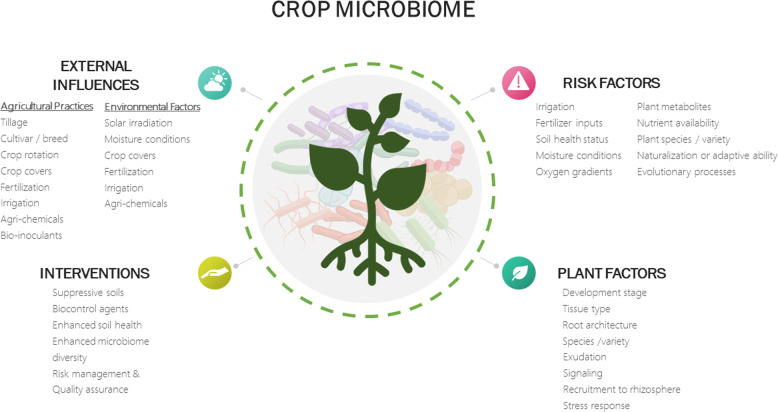
Schematic diagram. Schematic of the factors (plant, environmental and management) shaping the crop microbiome, the risk factors impacting the introduction and establishment of foodborne pathogen hazards, and the potential interventions (over-and-above existing quality assurance and risk management schemes) for the reduction of risk.

Microbial communities associated with plants impact many aspects of plant health and development across their life cycle: playing critical roles in nutrient, vitamin, water and hormone supply, stress resistance, and defence against pests and diseases [25, 26]. Endemic microbes within the soil and plant microbiota can be antagonistic to pathogens through a range of adversarial interactions, e.g. competition, niche exclusion, predation and metabolic defence, which can be effective means of pathogen biocontrol [25]. A wide range of interacting and dynamic factors shape the plant microbiota, including environmental, host-related and edaphic factors (Fig. 1). The plant exerts a strong selective effect on its microbial communities, actively recruiting beneficial microorganisms and deflecting those that are deleterious through modifications to the microenvironment or by inducing an immune response [27]. These plant-microbe interactions are partly facilitated by an array of chemical signals, which include phytohormones, exudates and volatile organic compounds [26]. Agricultural practices directly and indirectly impact the physical, chemical and biological properties of the environment housing plant microbiota (see Box 1). Management strongly influences plant microbial composition and function in microhabitats. For example, nitrogen fertilisation reduces phyllosphere bacterial diversity in rocket and spinach [28], and genetic variety and plant development stage results in wholesale changes in taxonomic composition [29]. Application of herbicides have implications for transmission of antimicrobial resistance, from upregulation of efflux pumps within the microbiome [15], which is of interest both in terms of microbial interactions within the plant microbiome and the potential transmission into the food chain. Indeed the recently evolved isolate of *E. coli* O104:H4 associated with the large-scale outbreak from fenugreek sprouts encodes ESBL resistance that is transmissible via plasmid conjugation [30]. As our understanding of plant microbiome increases a number of key questions arise: (i) can we predict microbial community assembly within a given cropping system?; (ii) how is assembly influenced by agricultural practices?; and (iii) are there scenarios that are suppressive to human pathogens within the plant microbiome?

**Fig. 2:**
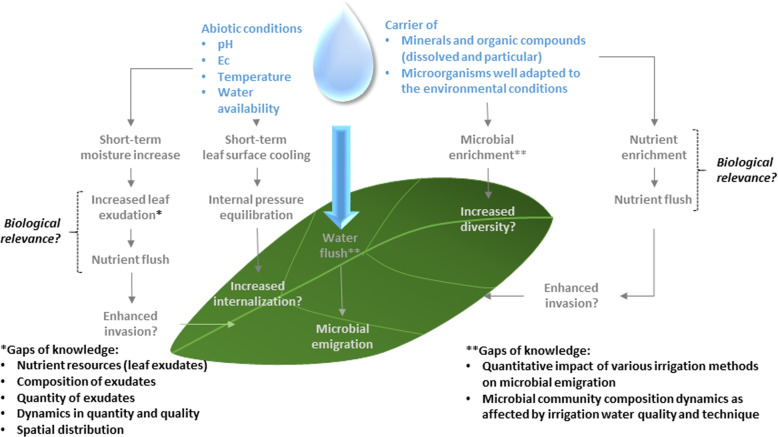
Schematic of the physio-chemical perturbations caused by canopy irrigation and the complexity of their potential impacts on the microbial community. Perturbations include resource availability and abiotic conditions. The potential impact on microbial community composition and function includes dynamics involving immigration, invasion, emigration, diversification and internalization. Invasion represents the potential success of alien organisms, such as human pathogens, in invading the microbial phyllosphere community. Gaps in knowledge and aspects where a better understanding of the biological relevance are needed are highlighted. EC electrical conductivity.

Prediction of microbial community responses to changes in agricultural practices, and what this means for pathogen suppression, is complex. Due to the inherent interdependencies, dynamic nature and complexity of plant-soil-microbial feedback mechanisms, the factors impacting microbial assembly cannot be presently ranked with certainty [31]. Importantly though, microbial assembly is known to be non-random and consistent patterns of core plant microbiome assembly have been observed, indicating hierarchical processes [32]. Critically therefore, if the source of microbiome components and the structuring principles are understood, prediction of the community under different agricultural practices is possible. However, to be effective, efforts at prediction need to go beyond microbial community structure to function [33] and consider the inherent physiological plasticity of the community in response to prevailing conditions.

In seeking to harness microbiome antagonism against human pathogens, much can be learned from efforts to control plant pathogens. Soil suppressiveness is influenced by the complex interactions between plants, soil, environment and microorganisms [34]. Plant community (especially diversity) mediated effects on soil abiotic and biotic properties have been identified as an important determinant [34]. Microbial interactions, including antagonism, are likely restricted to local hotspots because of the pronounced physical segregation of microniches associated with plants and localised access to resources [35]. Recently, some notable advancements in our understanding of microbiome suppression of plant pathogens have been reported, e.g. *Rhizoctonia solani* infection of sugar beet [36]. However, the process of identifying suppressiveness is inherently more difficult with human pathogens, since colonisation does not elicit a disease phenotype in the plant. Also challenging is that low concentrations of human pathogens on plants, often below detection limits, can still result in human infection. Finding a suppressiveness phenotype by chance without large-scale screening or targeted experimental testing of antagonistic effects is unlikely. Another uncertainty is the extent of both perception and control of human pathogens by plant host immunity. Plants can recruit specific microbial groups from soil to help suppress infections but this may not occur if a negative phenotype is not expressed in the plant host. Much will depend on the nature of the pathogen, its ecological interactions within the plant, and the mechanisms it employs to facilitate establishment.

Box 1 Case study: role of irrigation in shaping the plant microbiome Agricultural practices, such as irrigation, have the potential to directly or indirectly shape the plant microbiome but few studies have investigated the impact that water type, irrigation system or disinfection approaches exert on the plant microbiome. Allard et al. [56]. studied the impact of creek water irrigation on fresh produce microbiota (kale and radish), observing that irrigation influenced crop-associated microbiota, mainly in radish. Other studies showed that, although the microbial composition of irrigation water differed between water sources, irrigation of plants with different water sources did not significantly affect the bacterial composition of vegetables [57, 58]. Williams et al. [53]. showed that the bacterial community of romaine lettuce was affected by the irrigation system, observing that *Xanthomonas* was more abundant on sprinkler-irrigated lettuces, while *Pectobacterium*, *Leuconostoc* and *Lactococcus* were more frequent on drip-irrigated plants. No single genus consistently distinguished the microbiota between drip and sprinkler irrigated plants. Disinfection treatments can be applied to improve the microbiological quality of irrigation water, but its impact on the soil, plant and water microbiome is poorly understood. Truchado et al. [59]. evaluated the impact of chlorine dioxide (ClO_2_), as a water treatment for irrigation of baby spinach, and observed changes in the water microbiome but no significant differences in soil and plant microbiomes. Small changes were detected in the relative abundance of Pseudomonadaceae (14%) and Enterobacteriaceae (19%) in the plant, which is of interest due to the occurrence of potential pathogenic strains within these families. Another important finding is that the plant microbiota was more strongly influenced by the soil communities rather than those of irrigation water. While the relevance of irrigation water as a source of human pathogen contamination of crops is well documented [40], studies demonstrate, in general, that irrigation may not be the predominant factor shaping the plant microbiome. However, this needs to be assessed within the context of both biotic and abiotic drivers. A conceptual approach disentangling various factors underlying the impact of irrigation on the plant microbiome are displayed in Fig. 2.

## Ecological aspects of pathogen biocontrol

Pathogens that are passively transmitted on plant surfaces (e.g. viruses, protozoa), and require human or animal hosts for proliferation, are unlikely to be subject to suppressive interactions. However, in contrast there is strong evidence for active interactions between bacterial foodborne pathogens and plant hosts: plants actively perceive the bacteria; some pathogens mount a counter defence to host immunity; they form colonies and biofilms on plants (especially within the rhizosphere and on root surfaces), and can internalize and persist endophytically [12, 37]. For these pathogens that become established within the plant microbiome, including our most important foodborne pathogens, ecological concepts can be employed to understand the likelihood of pathogen establishment and the antagonistic potential of the endemic community. While empirical data on human pathogen-plant microbiome interactions remain scarce, ecological aspects applying to bioinoculant or invasive species in plant microbiomes are relevant. Both stochastic and niche-based processes shape the plant microbiome, although their relative importance is unclear [38]. Primarily, community dynamics are determined by dispersal, selection, drift and diversification processes, with microorganisms being added by speciation and dispersal, and thereafter being shaped by selection and drift [39]. Introduction of these ‘invaders’ to the community can occur through a breakdown of dispersal barriers by means of one, or recurring, contamination events that promote the presence or proliferation of pathogens, for example irrigation with contaminated water [40, 41]. For zoonotic pathogens these contamination events are considered the primary transmission route and a key mitigation target [40]. For other human pathogens that are frequently found in the environment, including as naturalised populations, such dispersal is not a limitation. Once within the community a key question is what properties of the pathogen enhance its capacity to establish? Initially, the likelihood of establishment may relate to the population size and diversity, as increased numbers provide insurance against stochastic events that could result in pathogen extinction [39, 42]. As selective pressures are exerted, the probability of pathogen establishment relates to its fitness for that environment.

Ecological concepts can also help predict the antagonistic potential of the endemic community. To establish themselves, pathogens need to avoid negative interactions resulting in extinction, and occupy ecological niches that enable population maintenance [43]. Microbial competition can be direct, whereas differences in metabolic requirements or metabolic flexibility circumvents competition to some extent. For example, STEC can switch to fatty acid degradation of root exudates when carbon availability is low [22]. Niche overlap between the pathogen and endemic population provides a greater likelihood of negative interactions and prevents coexistence. Bacterial nutrient resource utilization profiles can estimate ecological similarity of the pathogen and the resident community [44]. Dissimilar endemic communities with greater complementary and niche overlap are less susceptible to invasion. Both species richness and genomic dissimilarity of resident communities have been used as proxies for assessing ability to compete with an invader [39]. Genomic dissimilarity has been shown to determine allelopathic interactions impacting invasions by fostering competitive interactions [45]. For a negative interaction to occur the pathogen needs to overlap in space and time with the antagonistic agent, so the timing of pathogen introduction is important. Maximum impact is likely when the plant microbiome is well established prior to pathogen entry into the system (priority effect).

From an agronomic perspective this may mean that plants at the early growth stages are more vulnerable to pathogen establishment. However, dynamic change can occur at any stage in the plant life cycle, potentially destabilizing the endemic microbial network and allowing opportunity for pathogen establishment. Disturbances can lower microbial abundance and diversity, increasing the potential for pathogen emergence [39, 41]. Maximising diversity can act as an insurance policy, increasing functional redundancy and mitigating pathogen establishment opportunities caused by dynamic change or stress response [39]. Based on our current ecological understanding, agricultural practices that reduce disturbance and increase diversity, genetic richness and niche occupancy/overlap among endemic communities will increase antagonism against human pathogens. Agricultural practices impacting microbial diversity and richness in soil, as the main source of the plant microbiome, are particularly important, for example practices that deplete soil organic matter or contribute to reduced plant diversity, erosion, over-fertilization and compaction are likely to increase pathogen establishment risk. How best to assess diversity in the context of suppressive interactions needs to be carefully considered. While phylogenetic diversity is more readily determined, ultimately, the probability of pathogen suppressive interactions occurring is a function of the phenotypic diversity of the plant microbiome within a microniche.

## Microbiome-human pathogen interactions

While the factors influencing pathogen persistence in the plant environment are well understood [46, 47], there is limited understanding of exactly how plant or soil microbial communities negatively influence pathogens survival, whether this influence can be modulated, and what species are predominantly responsible for this effect. Some key considerations in understanding and utilising this effect are outlined in Box 2. Studies of disease suppression in plant production have focused on plant pathogens, and assessments of microbiome datasets to investigate functional links between agricultural practices, specific microbial groups and community composition are scant. However, a suppressive effect of *Pseudomonas fluorescens* strains on STEC and *Salmonella* has been demonstrated in soil and on produce [48, 49]. Conversely, it has been shown that certain phytobacteria promote human pathogens’ survival on plants [50]. The phenomenon of persister cell formation by human pathogens is also relevant, with a recent study demonstrating its occurrence in STEC on lettuce and in plant production relevant conditions [51]. This may contribute to residual pathogen populations on edible plants and raises the possibility of enhanced adaptation. How such sub-populations may differ in their interactions with native microbial communities is unknown. Recent studies indicate that specific agricultural practices (including cover cropping and compost addition) increased microbial suppression of pathogens in soils and that widely implemented agricultural practices (such as clearing of noncrop vegetation), aimed at reducing risk, may in fact increase pathogen prevalence by decreasing ecological interactions [10, 11, 52]. Investigation of the impact of human pathogens on the endemic microbiome in horticultural settings has yielded variable outcomes [53–55], indicating there are unlikely to be common rules, and that different plant holobiont systems need to be considered to assess the impact of ecological interactions. Adoption of contemporary microbiome analysis and network interactions will undoubtedly lead to new revelations in ecological interactions. Ideally, these interactions should be studied in real world conditions, where natural contamination has occurred, taking management, environmental and plant factors into account. Given the incidence of these pathogens on produce, and the contamination levels, such studies would need to be extremely large and sequencing undertaken to a significant depth to address these complex interactions. Lately, some initiatives have been undertaken where commercial growing fields have been incorporated into large-scale studies with the aim of identifying both potential risk factors linked to the contamination of crop plants with human pathogens and microbial community interactions.

Box 2 Key aspects to consider towards harnessing microbiomes for increased suppressiveness against human pathogens **Key aspects to consider**

- Suppressiveness is likely to be a function of the community as a whole rather than individual microbiota
- Some human pathogens may be part of normal plant microbiome
- Due to interdependencies, microbial interactions are likely to be context and pathogen-specific, making generic conclusions difficult
- Suppressive capacity within the community may not be expressed continually, and functional response could alter with prevailing conditions. This underlines the importance of microbial diversity to underpin functional resilience and the importance of community plasticity both pre- and post-harvest in the phenotypic outcome
- The selection or breeding of plants with reduced vulnerability to invasion by human pathogens is a possible mitigation approach. However, it must be considered in the context of wider microbe-plant interactions and other plant phenotypic characteristics
**Required research approaches**
- A functional approach that determines the role of each microbial component in facilitating or preventing pathogen establishment is needed
- Given the inherent spatial segregation and functional plasticity of the microbiota, an approach that determines what microbial components are interacting is needed
- Simpler models such as synthetic communities may clarify underlying ecological mechanisms but reductionist experimental approaches, looking at single elements, are unlikely to be helpful in elucidating complex system interactions
- Conditions facilitating the expression of suppressiveness need to be elucidated
- Multidisciplinary approaches, including relevant knowledge on plant physiology, agronomy, agricultural microbiota, and the food chain are needed to develop strategies to investigate functional interactions
- Combining genomic approaches, network analysis, dynamic monitoring, plant-microbe signalling analysis, and ecological modelling will enable better prediction of functional potential, ecological niches occupied, and how microbes interact with each other and the plant

## Conclusions

Management practices play a central role in shaping microbiomes in horticultural systems. Human pathogens possess fitness for occupying plant niches, posing a risk of foodborne illness in edible plants. Endemic microbial communities could effectively suppress human pathogens through antagonistic interactions, opening up the possibility of harnessing agricultural microbiomes to reduce disease transmission risk. Many interacting factors influence microbiome assembly and function, making it difficult to predict context-specific responses to agricultural activities and to translate these into practical interventions to increase microbiome suppressiveness. Studies that assess agricultural practice impacts on human pathogen–microbiome interactions and identify underlying mechanisms of suppression remain rare. However, the non-random nature of microbial assembly on plants indicates structuring principles exist that, once elucidated, should facilitate prediction of the plant microbiome. This, combined with recent success stories where communities suppressive to plant pathogens have been identified, offers hope of identifying microbial community contexts suppressive to human pathogens. As our understanding of the factors shaping microbial assembly, plant-microbe interactions and microbial function in plant systems is enhanced, ecological engineering of suppressive microbiomes via agricultural practice represents a realistic prospect. While nuanced engineering of plant microbiomes remains some way off, current ecological knowledge indicates agricultural practices supporting diverse microbiomes and reducing disturbance offer the best opportunity of preventing establishment of human pathogens in plant production systems.
